# ProteinMAE: masked autoencoder for protein surface self-supervised learning

**DOI:** 10.1093/bioinformatics/btad724

**Published:** 2023-11-29

**Authors:** Mingzhi Yuan, Ao Shen, Kexue Fu, Jiaming Guan, Yingfan Ma, Qin Qiao, Manning Wang

**Affiliations:** Digital Medical Research Center, School of Basic Medical Sciences, Fudan University, Shanghai 200032, China; Shanghai Key Laboratory of Medical Image Computing and Computer Assisted Intervention, Fudan University, Shanghai 200032, China; Digital Medical Research Center, School of Basic Medical Sciences, Fudan University, Shanghai 200032, China; Shanghai Key Laboratory of Medical Image Computing and Computer Assisted Intervention, Fudan University, Shanghai 200032, China; Digital Medical Research Center, School of Basic Medical Sciences, Fudan University, Shanghai 200032, China; Shanghai Key Laboratory of Medical Image Computing and Computer Assisted Intervention, Fudan University, Shanghai 200032, China; Digital Medical Research Center, School of Basic Medical Sciences, Fudan University, Shanghai 200032, China; Shanghai Key Laboratory of Medical Image Computing and Computer Assisted Intervention, Fudan University, Shanghai 200032, China; Digital Medical Research Center, School of Basic Medical Sciences, Fudan University, Shanghai 200032, China; Shanghai Key Laboratory of Medical Image Computing and Computer Assisted Intervention, Fudan University, Shanghai 200032, China; Digital Medical Research Center, School of Basic Medical Sciences, Fudan University, Shanghai 200032, China; Shanghai Key Laboratory of Medical Image Computing and Computer Assisted Intervention, Fudan University, Shanghai 200032, China; Digital Medical Research Center, School of Basic Medical Sciences, Fudan University, Shanghai 200032, China; Shanghai Key Laboratory of Medical Image Computing and Computer Assisted Intervention, Fudan University, Shanghai 200032, China

## Abstract

**Summary:**

The biological functions of proteins are determined by the chemical and geometric properties of their surfaces. Recently, with the booming progress of deep learning, a series of learning-based surface descriptors have been proposed and achieved inspirational performance in many tasks such as protein design, protein–protein interaction prediction, etc. However, they are still limited by the problem of label scarcity, since the labels are typically obtained through wet experiments. Inspired by the great success of self-supervised learning in natural language processing and computer vision, we introduce ProteinMAE, a self-supervised framework specifically designed for protein surface representation to mitigate label scarcity. Specifically, we propose an efficient network and utilize a large number of accessible unlabeled protein data to pretrain it by self-supervised learning. Then we use the pretrained weights as initialization and fine-tune the network on downstream tasks. To demonstrate the effectiveness of our method, we conduct experiments on three different downstream tasks including binding site identification in protein surface, ligand-binding protein pocket classification, and protein–protein interaction prediction. The extensive experiments show that our method not only successfully improves the network’s performance on all downstream tasks, but also achieves competitive performance with state-of-the-art methods. Moreover, our proposed network also exhibits significant advantages in terms of computational cost, which only requires less than a tenth of memory cost of previous methods.

**Availability and implementation:**

https://github.com/phdymz/ProteinMAE.

## 1 Introduction

Protein surface is a high-level representation of protein, which encodes the external structures involved in interaction with molecules and is highly correlated with the properties of the protein. The representation of protein surface is widely used in applications such as protein–protein interactions prediction (Planas-Iglesias *et al.* 2013, Wang *et al.* 2017) and drug design (Venkatraman *et al.* 2009, Daberdaku and Ferrari 2019, Gainza *et al.* 2023). Traditional methods (Yin *et al.* 2009, Kihara *et al.* 2011) rely on complex expertise and manual modeling to construct surface descriptors, which commonly have limited representation capabilities and task dependencies. In recent years, with the booming advance of deep learning (Berrar and Dubitzky 2021), a series of learning-based methods (Gainza *et al.* 2020, Sverrisson *et al.* 2021) have been proposed and achieved inspiring performances in protein representation.

However, these learning-based methods are highly reliant on training data. Insufficient training data tend to cause overfitting, resulting in limited performance. In the case of protein representation learning, the acquisition of labeled data such as protein properties is challenging and expensive, because the labels are often obtained through wet experiments. Although the labeled data are scarce, unlabeled data such as raw protein structures (Berman *et al.* 2000) are much richer, inspiring us to explore the self-supervised learning (Chen *et al.* 2020, He *et al.* 2020) for protein representation.

Self-supervised learning is already an option in the fields of natural language processing (Devlin *et al.* 2018, Liu *et al.* 2023) and computer vision (Bao *et al.* 2021, Chen and He 2021) for addressing the lack of labeled data. It typically follows a “pretraining and then fine-tuning” paradigm, where the model is first pretrained on unlabeled data through a proxy task and then fine-tuned on labeled data for downstream tasks. The pretrained weights initialization enables the model to achieve better performance on downstream tasks. Self-supervised learning has demonstrated strong performance in various fields and also has potential in protein surface representation learning, but existing methods (Gainza *et al.* 2020, Sverrisson *et al.* 2021) are not optimal for self-supervised learning despite their outstanding performance in protein surface representation learning. For example, MaSIF (Gainza *et al.* 2020) represents protein surfaces in the form of meshes and utilizes geodesic convolution to extract surface descriptors. This leads to complex preprocessing including mesh construction and geodesic distance calculation, making pretraining on large-scale datasets unaffordable. dMaSIF (Sverrisson *et al.* 2021) represents protein surfaces in the form of point clouds and utilizes an efficient quasigeodesic distance, which successfully avoids complex preprocessing (Kyte and Doolittle 1982, Sanner *et al.* 1996, Jurrus *et al.* 2018). However, its geometric convolutional neural network has a quadratic complexity of point number, making the cost of inference unaffordable especially when using a large batch size. Therefore, to achieve self-supervised learning for protein surface representation learning, a more efficient network is needed.

In this article, we propose a framework named ProteinMAE to tackle above challenges. Specifically, we represent protein surfaces as point clouds, which enable us to implement pretraining on a large-scale dataset without complex preprocessing. Based on these protein surface point clouds, we propose an efficient transformer-based (Vaswani *et al.* 2017) network for protein surface representation learning, which only requires 1/10 memory of dMaSIF and 1/100 memory of MaSIF. Our network is inspired by the great success of transformer-based networks (Han *et al.* 2022) in computer vision like ViT (Dosovitskiy *et al.* 2020). It divides protein surface point clouds into patches and uses a transformer to extract features based on inter-patch dependencies, rather than inter-point dependencies as dMaSIF, significantly reducing memory usage. Moreover, we believe that chemical properties play an important role in protein representation learning, so our network not only encodes geometric features similar to some point cloud networks (Pang *et al.* 2022) but also incorporates the encoding of chemical properties. Based on our efficient network, we can successfully conduct a Masked AutoEncoder-style (He *et al.* 2022) proxy task on large-scale unlabeled data. In our proxy task, we randomly mask a portion of the point clouds and predict the masked portion based on the chemical and geometric features of the remaining unmasked portion. This task not only enables easy implementation on the large-scale dataset but also requires no manual annotation. After pretraining on a large-scale dataset, we utilize the pretrained weights to initialize the network and fine-tune it on downstream tasks. Benefiting from the self-supervised learning on the large-scale dataset, our network exhibits better performance on the downstream tasks than training from scratch.

To evaluate the effectiveness of our method, we conduct experiments on three downstream tasks including binding site identification in protein surface, ligand-binding protein pocket classification, and protein–protein interaction prediction. Our self-supervised learning successfully enhances the network’s performance on all downstream tasks and enables it to achieve competitive performance with state-of-the-art methods.

Overall, our contributions can be summarized as follows:

- We propose ProteinMAE, a self-supervised learning framework for protein surface representation learning, which utilizes a large amount of accessible unlabeled data to boost performance on downstream tasks.
- We propose an efficient network suitable for self-supervised learning, which has a competitive performance to previous work but with less than a tenth of the memory cost.
- Benefiting from pretraining on large unlabeled data, our network can achieve improvement on all downstream tasks and new state-of-the-art performance in some tasks.

## 2 Related work

### 2.1 Protein surface representation learning

Protein surface plays an important role in applications such as drug design (Kihara *et al.* 2011, Zhu *et al.* 2015, Daberdaku and Ferrari 2019, Gainza *et al.* 2023), as it directly involves the interaction between proteins and other molecules. The representation of protein surface can be used in describing the properties of proteins and other tasks such as docking. Traditional methods (Kihara *et al.* 2011, Zhu *et al.* 2015, Daberdaku and Ferrari 2019) of protein surface representation rely on complex expertise and manual modeling, which often have limited performance. Recently, inspired by the booming development of geometric deep learning, MaSIF (Gainza *et al.* 2020), as a pioneer, first introduces geometric deep learning to protein surface representation. It designs a geodesic convolution which can extract both geometric and chemical features from the protein surface mesh, surpassing a series of traditional methods. However, the triangulation and precomputation of geodesic patches cause a large computational overhead. To tackle this problem, dMaSIF (Sverrisson *et al.* 2021) replaces mesh with point cloud to represent protein surface and proposes a quasigeodesic convolution to replace geodesic convolution. However, we find dMaSIF still incurs a relatively large computational overhead, as its quasigeodesic convolution exhibits quadratic complexity with respect to surface points. As self-supervised learning typically requires a large batch size, it is less ideal for MaSIF and dMaSIF to serve as the backbones for self-supervised learning. To address this problem, we propose a framework suitable for self-supervised learning. In our framework, the surface point clouds are divided into many patches and our network learns the dependencies between patches rather than the points, making our network have a competitive performance but with less than a tenth of the memory cost of MaSIF and dMaSIF.

### 2.2 Self-supervised learning

Typically, self-supervised learning aims at pretraining models through a proxy task without manually annotated labels. It can be coarsely divided into contrastive learning-based methods (He *et al.* 2020, Chen and He 2021) and reconstruction-based methods (He *et al.* 2022, Pang *et al.* 2022, Zhang *et al.* 2022) according to their proxy tasks. The former typically achieves pretraining by constructing and distinguishing positive and negative samples, while the latter achieves pretraining by predicting the unobserved parts. Over the past few years, self-supervised learning has been widely used in natural language processing (Devlin *et al.* 2018, Liu *et al.* 2023) and computer vision (Chen and He 2021, He *et al.* 2022), substantially enhancing models’ performance across many downstream tasks. However, research on protein surface has seldom applied self-supervised learning, although the label scarcity problem also exists in protein-related research. The reason may be that the very large computational overhead of existing methods (Gainza *et al.* 2020, Sverrisson *et al.* 2021) limits the application of self-supervised techniques for protein surface learning. In this article, we propose a lightweight reconstruction-based framework to achieve self-supervised learning for protein surface representation learning. Moreover, we also incorporate a contrastive learning-based competitor to demonstrate the effectiveness of our method. Our experiments show that the proposed method not only successfully improves the network’s performance on all downstream tasks, but also outperforms the self-supervised learning method based on contrastive learning.

## 3 Materials and methods

**Figure 1.**
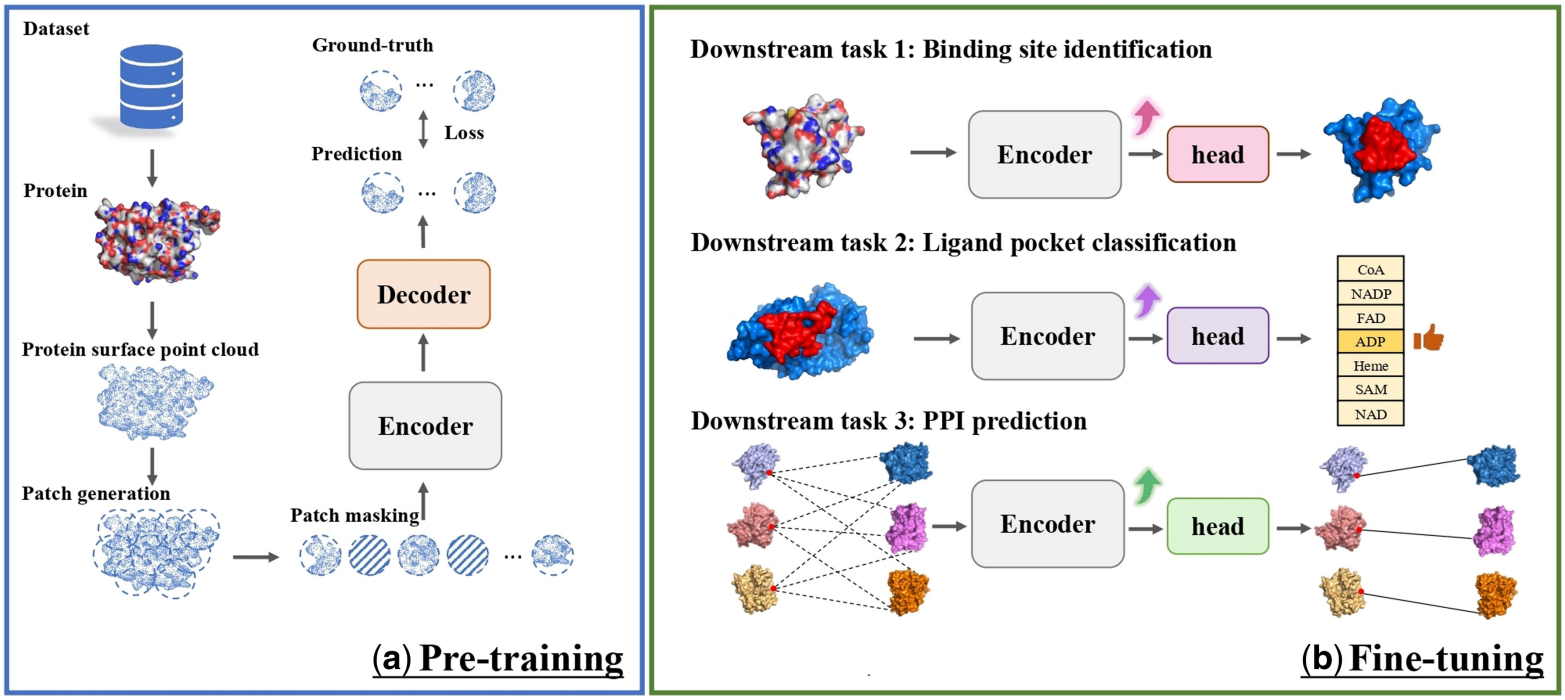
Overview of ProteinMAE. (a) We execute a masked reconstruction proxy task to pretrain our network; (b) We initialize the encoder using the pretrained weights and retrain the network for different downstream tasks. Benefiting from pretraining on large unlabeled data, our network can achieve improvement on all downstream tasks. Binding site identification: The network receives a protein as input and outputs a surface score that reflects the predicted interface propensity; Ligand pocket classification: The network receives a protein pocket as input and categories it into seven distinct classes, thereby illustrating the predicted binding preference; PPI prediction: The network receives proteins as input and generates descriptors for them to measure the probabilities of their binding.

In this section, we will introduce our proposed method ProteinMAE. As shown in Fig. 1, ProteinMAE consists of two phases: pretraining and fine-tuning. In the pretraining phase, a masked reconstruction proxy task is executed to learn the initialization weights of the backbone network. In the fine-tuning stage, we initialize the encoder using the pretrained weights and retrain it for different downstream tasks, for each of which a specific head is utilized. We will describe the detail of ProteinMAE in the following sections.

### 3.1 Data preparation

As mentioned before, a wealth of unlabeled protein data is available, with the prominent Protein Data Bank (PDB) (Berman *et al.* 2000) containing around 190 000 protein structures. To prepare our data for pretraining, we downloaded 190 615 proteins from PDB. Later, recognizing that most downstream tasks do not fully concern the complete structures of proteins, we decompose each of the downloaded proteins into multiple chains to obtain more data for pretraining, while keeping the proteins that consist of a single chain unchanged. This process yields 620 364 protein chains. Additionally, to eliminate the interference from minority class atoms and save computational cost, we remove the chains containing atoms other than {C, H, O, N, S, Se} (constituting over 99.9% of the total atomic composition). As a result, we obtain a total of 359 255 protein chains for pretraining. Each protein chain obtained is represented by a set of atom coordinates ${\left\{a_{i}\right\}}_{i=1}^{M}\in R^{M\times 3}$ and the corresponding atom types ${\left\{t_{i}\right\}}_{i=1}^{M}\in R^{M\times 6}$, where $a_{i}\in R^{1\times 3}$ represents the atom coordinate of the *i*-th atom in the protein chain, which has been centered, and $t_{i}\in R^{1\times 6}$ represents the one-hot encoding of the atom types. Next, we construct the protein surface point clouds and calculate the corresponding chemical and geometric properties following the process in dMaSIF (Sverrisson *et al.* 2021). Specifically, for each protein chain, we use a smooth distance function (Blinn 1982) and the van der Waals radii of the atoms to obtain an approximate modeling of the protein surface, which is then sampled at a resolution of 1 Å to obtain a point cloud ${\left\{x_{i}\right\}}_{i=1}^{N}\in R^{N\times 3}$ of the protein surface, where $x_{i}\in R^{1\times 3}$ represents the coordinate of the *i*-th constructed surface point, *N* represents the number of points of the surface point cloud. The protein chains with N>20,000 are filtered out, resulting in 356 039 protein surface point clouds available for pretraining. Furthermore, we calculate simple but effective geometric and chemical features for each point in the surface point clouds. We utilize the gradient of the smooth distance function to obtain the normal vector $n_{i}\in R^{1\times 3}$ at each point $x_{i}\in R^{1\times 3}$ and utilize normals and coordinates to further calculate the corresponding mean and Gaussian curvatures ${\left\{u_{i}\right\}}_{i=1}^{N}\in R^{N\times 10}$, where $u_{i}\in R^{1\times 10}$ denotes the mean and Gaussian curvatures (Cao *et al.* 2019) at the scales of 1 Å,2Å,3Å,5Å,10Å. For each point $x_{i}\in R^{1\times 3}$ in the surface point clouds, its mean and Gaussian curvatures $u_{i}\in R^{1\times 10}$ are considered as its corresponding geometric feature. We also calculate a raw chemical feature for each point, as Sverrisson *et al.* (2021) have demonstrated that chemical properties such as Poisson–Boltzmann electrostatic can be learned from the raw chemical features like atom type distribution. Given a point $x_{i}\in R^{1\times 3}$ in the surface point clouds, we gather its 16 nearest neighbor atoms and treat the distances and the atom types of the gathered atoms as the corresponding raw chemical feature for $x_{i}$. The raw chemical feature of point $x_{i}$ can be represented as $\{(t_{j},d_{ij})\;|\;a_{j}\in N_{i}\}\in R^{16\times 7}$, where $N_{i}$ denotes the neighborhood of the $x_{i}$, $a_{j}\in R^{1\times 3}$ denotes the atom coordinate in $N_{i}$, $t_{j}\in R^{1\times 6}$ denotes a one-hot atom type encoding of an atom in $x_{i}$’s neighbor, $d_{ij}\in R^{1\times 1}$ denotes the Euclidean distance between $x_{i}$ and $a_{j}$. These raw chemical features and geometric features are saved with the protein surface point clouds and will be further processed by a learnable module in the encoder, which will be detailed in Section 3.2.2. The above preprocessing is also applicable to our downstream tasks. Slightly different from the preprocessing for pretraining, the preprocessing for downstream tasks does not involve the decomposition of proteins, meaning we construct the surface point clouds and compute features for the complete proteins rather than the protein chains.

### 3.2 Framework of pretraining

After data preprocessing, the raw data of proteins have been transformed into protein surface point clouds and each point has a corresponding feature of dimension 122 (16×7+10). We conduct a proxy task, i.e. masked reconstruction to achieve self-supervised pretraining. As shown in Fig. 1a, each input protein surface point cloud is divided into numerous patches, some of which are masked as prediction targets, while the remaining patches are fed into the encoder. The encoder tokenizes the patches and extracts patch-level features from the geometric and raw chemical features within the patches, as well as dependencies between patches. The decoder takes patch-level features as input and predicts the masked patches. We will discuss the pretraining process in more detail in the following sections.

#### 3.2.1 Patches generation and masking

**Figure 2.**
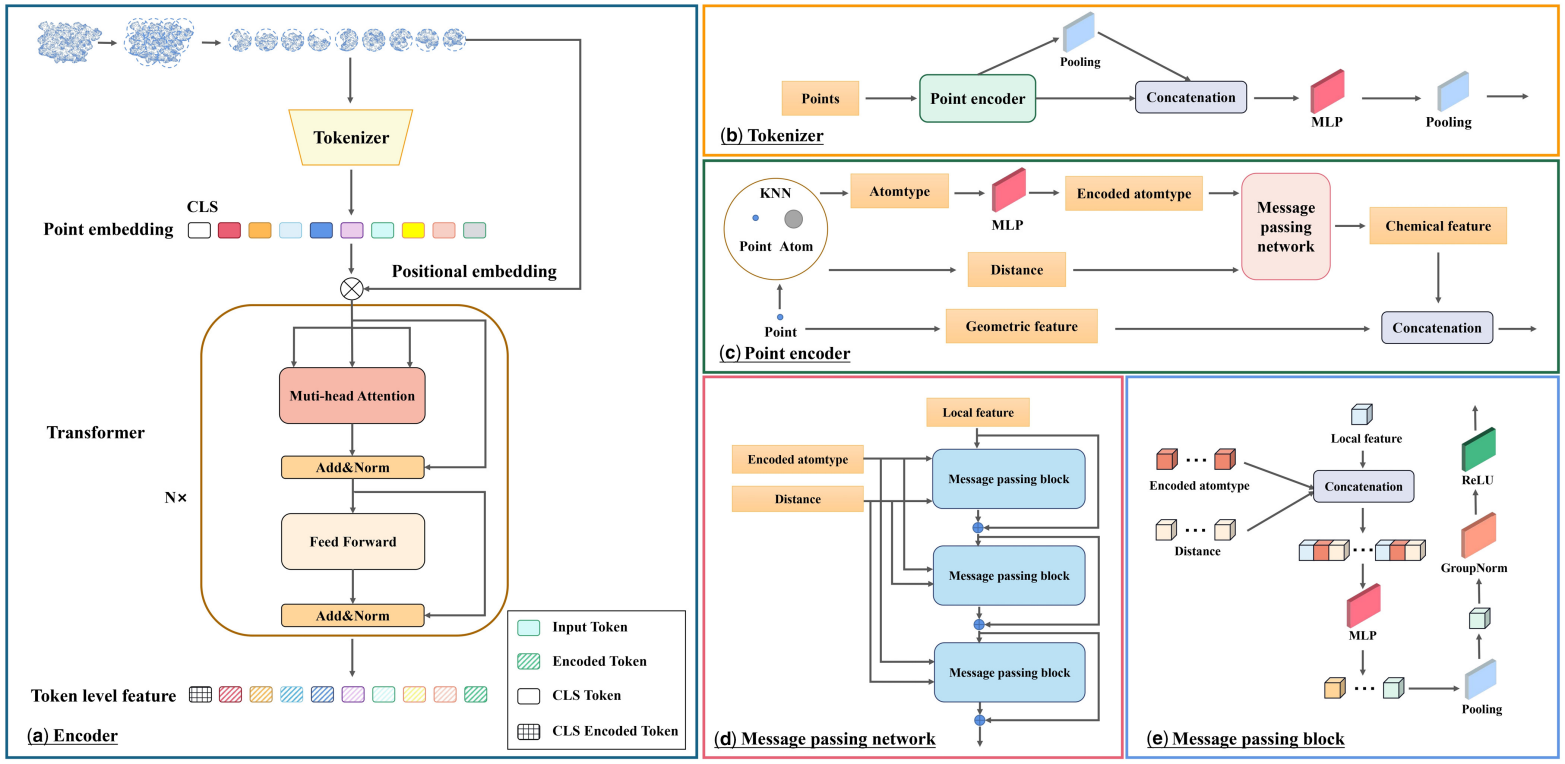
The detailed architecture of the encoder. We show the details of each component in a hierarchical progression. (a) Encoder: Given an input protein surface point cloud, we first divide it into patches and randomly mask a proportion of them. Then the unmasked patches are fed into the encoder. Within the encoder, a tokenizer is employed to convert the unmasked patches into tokens, enabling their further processing in the transformer. The following transformer will take tokens as input and extract features for downstream tasks or reconstruction in pretraining. (b) Tokenizer: Our tokenizer takes points within patches as input and utilizes a point encoder to extract point-level feature for each point. These extracted features are further processed by a MLP and are subsequently fused through a max-pooling layer to generate features for patches. (c) Point encoder: The point encoder in tokenizer takes each point within the patches as input and further processes the raw chemical features. The chemical features including atom types of neighbor atoms and their corresponding Euclidean distances are processed into 6D features through a MLP and a message passing network. The processed chemical features are then concatenated with the precalculated geometric features and collectively output. (d and e) Message passing network: The message passing network consists of three message passing blocks. Each block takes a local feature, encoded atom types of neighbor atoms and their corresponding distances as input and output a local feature for further extraction. The initial local feature is set to an all-one vector.

As shown in Fig. 2a, our encoder contains a tokenizer and a transformer (Vaswani *et al.* 2017). The tokenizer transfers the input protein surface point clouds into tokens. The transformer takes tokens as inputs and uses self-attention blocks to extract features for subsequent reconstruction. Since the self-attention has quadratic complexity, it is impossible to treat each point in the surface point cloud as a token. Therefore, inspired by ViTs (Dosovitskiy *et al.* 2020) approach of processing images as patches, we also divide the protein surface point cloud into several patches based on the coordinates of the points. Specifically, given an input point cloud, we first sample *g* center points using farthest point sampling (FPS), which are also the center points of *g* patches. Then, we find $k^{\prime }$ nearest neighbor points for each center point using KNN, partitioning the input point cloud into irregular and possibly overlapping patches ${\left\{P_{i}\right\}}_{i=1}^{g}=\text{KNN}\left({\left\{c_{i}\right\}}_{i=1}^{g},{\left\{x_{j}\right\}}_{j=1}^{N}\right)\in R^{g\times k^{\prime }\times 3}$, where $P_{i}\in R^{k^{\prime }\times 3}$ denotes the *i*-th patch in the point cloud, ${\left\{c_{i}\right\}}_{i=1}^{g}\in R^{g\times 3}$ denote the sampled center points. Before feeding patches into the transformer, we randomly mask a certain proportion of the patches for subsequent reconstruction prediction. The mask ratio *m* is empirically set to 60%. In the subsequent experiments (details in Section 4.5), we will also discuss in detail the effects of different mask ratios on downstream task performance. During fine-tuning and inference, we will no longer mask any patches.

#### 3.2.2 Encoder architecture

As shown in Fig. 2a, our encoder consists of a tokenizer and a transformer. The tokenizer is designed to convert the patches into a series of tokens. During this process, the raw chemical features obtained in the data preprocessing stage are further processed by the tokenizer. Specifically, we have obtained the raw chemical features, including distances and types of the neighbor atoms for each point. The tokenizer utilizes a MLP and three message passing layers to encode these information and outputs a 6D feature $f_{i}^{c}\in R^{1\times 6}$ to represent the local chemical feature for each point. Afterward, the local chemical feature $f_{i}^{c}\in R^{1\times 6}$ is concatenated with the precalculated geometric features $u_{i}\in R^{1\times 10}$ as the new feature $f_{i}^{\mathrm{cat}}\in R^{1\times 16}$ for each point.

Each unmasked patch then gathers the concatenated features of all points within the patch and uses a simple MLP and pooling layer to convert the patch into a series of *d*-dimension token embeddings ${\left\{f_{i}\right\}}_{i=1}^{(1-m)g}\in R^{(1-m)g\times d}$, where $f_{i}\in R^{d}$ denotes the token embedding for *i*-th patch.

Since the relative spatial position between protein structures has a significant impact on protein properties, position encoding is added to the unmasked tokens to introduce relative spatial position information. We use a learnable position encoding approach, i.e. an MLP that embeds the center coordinate of each patch into the embedding space. The position encoding is added to the token embedding. The following transformer takes features ${\left\{f_{i}+\text{MLP}\left(c_{i}\right)\right\}}_{i=1}^{(1-m)g}\in R^{(1-m)g\times d}$ as input and outputs the encoded features ${\left\{h_{i}\right\}}_{i=1}^{(1-m)g}\in R^{(1-m)g\times d}$ through multiple self-attention blocks for subsequent reconstruction.

In addition, we also incorporate a specialized token, i.e. the CLS Token in Fig. 2 for classification downstream tasks. This token consists of a sequence of learnable parameters and is input together with other tokens into the transformer for feature extraction, but not used as input to the decoder.

#### 3.2.3 Loss function

During the pretraining stage, our decoder takes the encoded features ${\left\{h_{i}\right\}}_{i=1}^{(1-m)g}\in R^{(1-m)g\times d}$ as input and predicts the point coordinates of the masked patches. Our decoder consists of four self-attention blocks and a linear prediction head. Given the predicted patches ${\left\{P_{i}^{\text{pred}}\right\}}_{i=1}^{mg}\in R^{mg\times 3}$ and the ground truth ${\left\{P_{i}^{\text{mask}}\right\}}_{i=1}^{mg}\in R^{mg\times 3}$, our reconstruction loss is a chamfer loss (Fan *et al.* 2017):

(1) $$\begin{array}{c} L=\sum_{i=1}^{mg}(\frac{1}{\left|P_{i}^{\text{mask}}\right|}\sum_{x\in P_{i}^{\text{mask}}}\underset{y\in P_{i}^{\text{pred}}}{\min}||x-y|{|}_{2}^{2}\\ +\frac{1}{\left|P_{i}^{\text{pred}}\right|}\sum_{x\in P_{i}^{\text{pred}}}\underset{y\in P_{i}^{\text{mask}}}{\min}||x-y|{|}_{2}^{2}), \end{array}$$

where $\left|P_{i}^{\text{pred}}\right|$ represents the number of predicted coordinates of *i*-th masked patches, and $\left|P_{i}^{\text{mask}}\right|$ represents the number of ground truth coordinates of *i*-th masked patches. During the pretraining stage, we select the optimal epoch based on the reconstruction loss and utilize the weights with minimal reconstruction loss for downstream tasks.

### 3.3 Fine-tuning on downstream tasks

As shown in Fig. 1b, our pretrained model can be fine-tuned on downstream tasks to enhance the network’s performance. The fine-tuning process involves using the weights of the pretrained encoder as initialization weights for the encoder, and then retraining the whole model for different downstream tasks based on their respective training data. We conduct three different downstream tasks including binding site identification in protein surface, ligand-binding protein pocket classification, and protein–protein interaction prediction. During fine-tuning on downstream tasks, we no longer mask any patches.

#### 3.3.1 Binding site identification in protein surface

Binding site identification aims at classifying the protein surfaces into interaction sites and noninteraction sites. Identifying the location of binding site is crucial for ligand binding, as small molecules only inhibit or activate specific biological functions by binding to specific protein pockets. Setting up the downstream task of predicting binding site can evaluate whether the pretrained network has learned geometric and chemical information on the protein surface. For this downstream task, we add a head to identify the binding site, which takes encoded features as inputs and outputs the point-level binary prediction. During fine-tuning, we use the same balanced cross-entropy loss as dMaSIF (Sverrisson *et al.* 2021). More details about the fine-tuning on this task can be found in Supplementary Material.

#### 3.3.2 Ligand-binding protein pocket classification

Given a protein pocket, ligand-binding protein pocket classification aims at estimating its binding preference to other molecules such as metabolites. Interactions between proteins and metabolites play a fundamental role in cellular homeostasis, but current understanding of these interactions is very limited. Our pretraining has learned the geometric and chemical properties of protein surfaces and can help to learn the metabolic-binding preferences of protein pockets. This downstream task is to predict the preferences of each protein for seven cofactors [ADP, NAD, NADP, FAD, S-adenosyl methionine (SAM), coenzyme A (CoA), and HEME]. As this downstream task is a classification task, we add a classification head for fine-tuning. The classification head takes the encoded features of the CLS Token and the pooled features of the other tokens as inputs and outputs the probabilities of affinity for the seven cofactors. We use a cross-entropy loss (Theodoridis and Koutroumbas 2006) for fine-tuning on this task.

#### 3.3.3 Protein–protein interaction prediction

The prediction of protein–protein interaction is essential in protein design tasks, as it serves as a starting point for protein docking. In this task, given two protein surface patches, we extract two descriptors for them, respectively. The similarity between the descriptors measures the probability of their binding. For this task, we utilize an identity network as our head, which is equivalent to directly using the outputs of the encoder as the descriptors. During fine-tuning, we use same balanced metric loss as dMaSIF (Sverrisson *et al.* 2021), which forces the features of patches that can bind close to each other in feature space, while those that cannot bind are far away.

### 3.4 Implementation

We utilize the Pytorch (Paszke *et al.* 2017) framework to implement our network. For pretraining, we optimize our network using an AdamW (Loshchilov and Hutter 2017) optimizer for 50 epochs. The initial learning rate is set to 0.001, and the batch size is set to 16. Furthermore, we set the number of protein surface point cloud to 2048 and default the mask ratio to 60%. We provide a comprehensive outline of downstream task implementation in Supplementary Material. All the experiments are conducted on a single RTX2080Ti graphic card.

## 4 Results and discussion

### 4.1 Binding site identification in protein surface

#### 4.1.1 Dataset

We utilize the dataset of binding site identification task in dMaSIF (Sverrisson *et al.* 2021), which consists of protein complexes extracted from the PDB (Berman *et al.* 2000). The dataset comprises 2958 proteins for training and 356 proteins for test, with 10% of the training set allocated for validation. The objective of this downstream task is to classify the protein surface into interaction or noninteraction sites, in other words, to predict binary labels at the point level.

#### 4.1.2 Competitors

We mainly compare our method with two advanced learning-based competitors, namely MaSIF (Gainza *et al.* 2020) and dMaSIF (Sverrisson *et al.* 2021). Since many traditional methods (Porollo and Meller 2007, Murakami and Mizuguchi 2010) cannot achieve competitive performance within an acceptable time, we do not consider them as competitors. Among our competitors, MaSIF is a mesh-based method, which extracts protein representation by conducting convolution on reconstructed protein surface mesh. It outperforms many traditional methods but still requires complex preprocessing including surface mesh reconstruction and chemical property calculation. dMaSIF is a more efficient version of MaSIF, which treats the protein surface as point cloud rather than mesh. It can achieve competitive performance but is free of complex preprocessing. Furthermore, we also incorporate two additional competitors to demonstrate the effectiveness of our pretraining method. The former involves training from scratch using the same network as ours, without leveraging pretrained weights for initialization. The latter, similarly employing the same network as ours, substitutes the masked reconstruction proxy task with a contrastive learning-based proxy task (Chen and He 2021).

#### 4.1.3 Evaluation metric

Following the setting in Sverrisson *et al.* (2021), we utilize the ROC-AUC (Theodoridis and Koutroumbas 2006) as our evaluation metric. Moreover, we also incorporate accuracy, recall, and F1 score (Theodoridis and Koutroumbas 2006) as our evaluation metrics to achieve a more comprehensive evaluation.

#### 4.1.4 Experimental results

**Figure 3.**
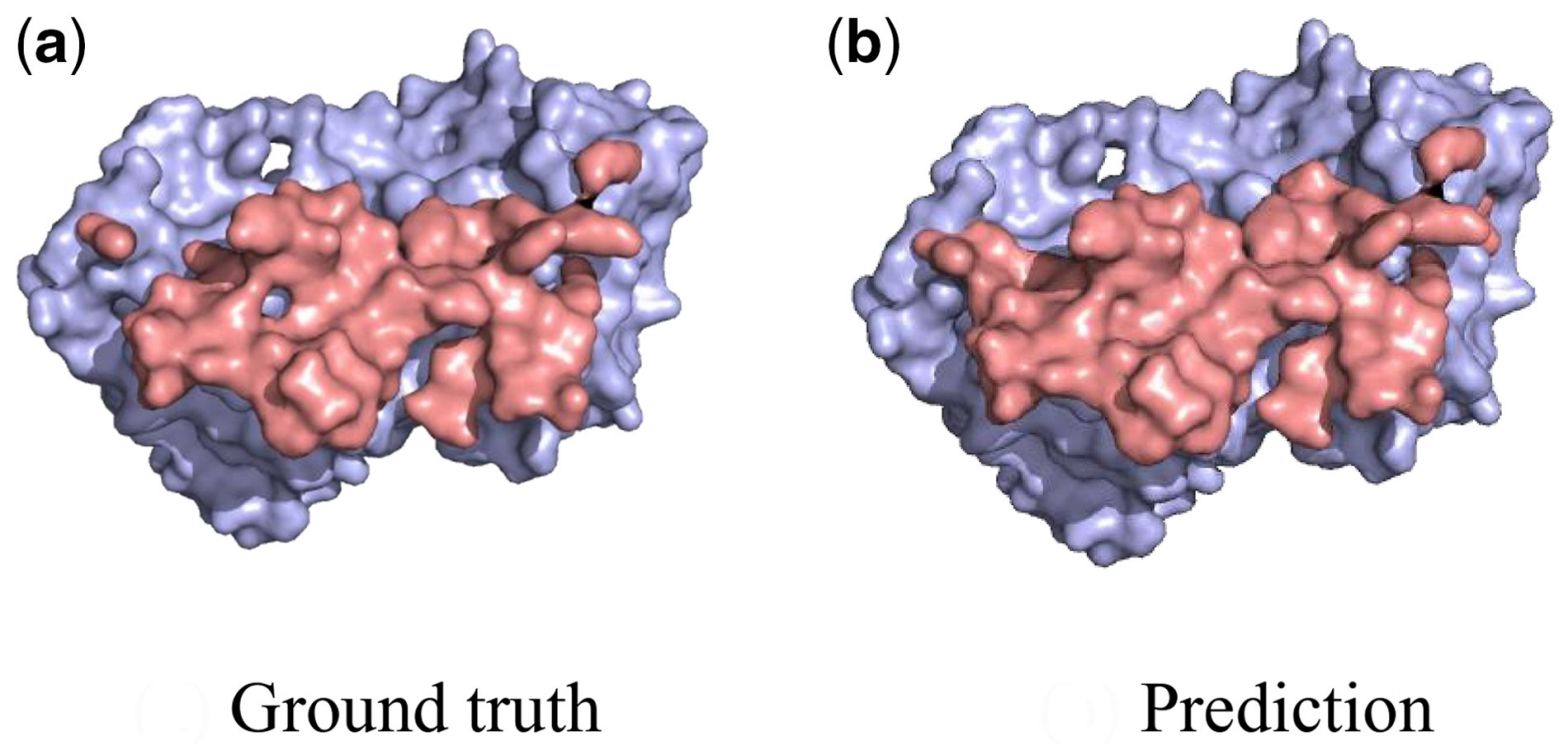
Visualization of our prediction on binding site. We utilize light red to represent the ground truth and light blue to represent our prediction. Our prediction is very close to the ground truth.

**Figure 4.**
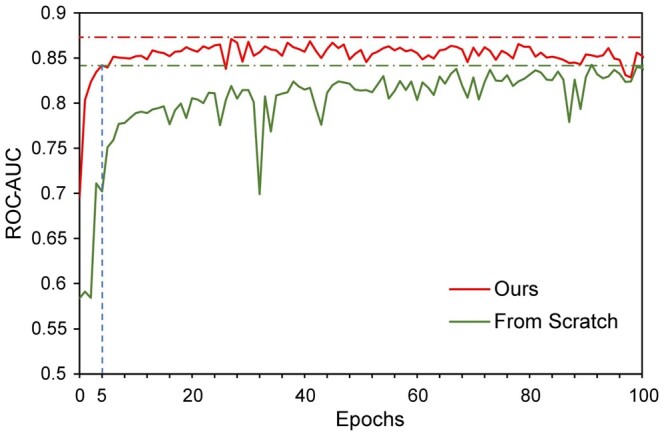
Convergence curve. We compare the performance of our transformer-based networks trained from scratch (green) and trained with pretrained weights as initialization (red) in terms of ROC-AUC on binding site identification downstream task. The red dotted line indicates the best performance of our method while the green dotted line indicates the best performance of the network trained from scratch. Our method reaches the best performance of the network trained from scratch in only five epochs.

We present the performance of our method and the competitors in Table 1. It can be observed that our method achieves the best performance on most evaluation metrics. We visualize some of our prediction results in Fig. 3, which demonstrate high accuracy. Our transformer-based network also demonstrates high competitiveness, achieving comparable performance to MaSIF (Gainza *et al.* 2020) even without pretraining. In addition, our transformer-based method has a significant advantage in computational cost, which will be further explained in the subsequent computational cost analysis. Compared to ours (from scratch), we can find that both the masked reconstruction proxy task and the contrastive learning-based proxy task enhance the performance, but the former enhances more, demonstrating the effectiveness of our ProteinMAE. Moreover, self-supervised learning can accelerate the convergence speed of the network on downstream task. We provide a convergence curve in Fig. 4, which shows that our method can reach the final performance of “training from scratch” in the 5-th epoch, significantly reducing the required cost for downstream task training.

**Table 1.** Performance on binding site identification.

Method	Accuracy↑	Recall↑	F1 score↑	ROC-AUC↑
MaSIF	0.741	**0.864**	0.760	0.847
dMaSIF	0.774	0.781	0.763	0.865
Ours (from scratch)	0.765	0.785	0.756	0.843
Ours (contrastive)	0.788	0.772	0.769	0.866
Ours	**0.793**	0.799	**0.782**	**0.871**

We use bold to highlight the optimal value.

### 4.2 Ligand-binding protein pocket classification

#### 4.2.1 Dataset

We utilize the dataset of ligand-binding pocket classification task in MaSIF (Gainza *et al.* 2020), which consists of 1459 structures. We follow the settings in Gainza *et al.* (2020) to use 72%/8%/20% data for training/validation/test. Each pocket in this dataset is classified into seven categories (ADP, NAD, NADP, FAD, SAM, CoA, and HEME) based on its affinity to metabolites.

#### 4.2.2 Competitors

Similar to the binding site identification task, we utilize dMaSIF (Sverrisson *et al.* 2021) and MaSIF (Gainza *et al.* 2020), our backbone trained from scratch, as well as our backbone pretrained by contrastive learning (Chen and He 2021) as competitors. Since dMaSIF is not originally designed for the ligand-binding protein pocket classification task, we modify it and fully tune it on this task. Specifically, we utilize a global max pooling layer to aggregate point-level features, followed by a multilayer perceptron to classify the aggregated global features.

#### 4.2.3 Evaluation metric

Since the category distribution of the dataset is not exactly balanced, we utilize the balanced accuracy (Mower 2005) as our evaluation metric. It takes into account not only the overall accuracy of the model but also its ability to correctly classify samples from underrepresented classes.

#### 4.2.4 Experimental results

We present our method and competitors’ performance in Table 2, where the results of MaSIF are derived from the original paper. Our method does not outperform MaSIF. This is because typically meshes are more advantageous for classification tasks compared to point clouds (Hu *et al.* 2022). Our proposed transformer-based model performs better than dMaSIF, which also uses point clouds to represent protein surfaces. Our pretraining approach successfully improves the performance of our transformer-based model from 0.666 to 0.707, while contrastive learning only improves marginally, which demonstrates the effectiveness of our ProteinMAE. We provide analysis on feature distribution with and without self-supervised learning in Supplementary Material.

**Table 2.** Performance on ligand-binding pocket classification.

Method	Balanced accuracy↑
MaSIF	**0.74**
dMaSIF	0.623
Ours (from scratch)	0.666
Ours (contrastive)	0.667
Ours	0.707

We use bold to highlight the optimal value.

### 4.3 Protein–protein interaction prediction

#### 4.3.1 Dataset

We utilize the dataset of protein–protein interaction prediction task in dMaSIF (Sverrisson *et al.* 2021), which consists of 5526 protein complexes. We follow the settings in Sverrisson *et al.* (2021) to use 4614 data for training and 912 data for test. Additionally, we allocate 10% of the training set for validation.

#### 4.3.2 Competitors

We utilize the same competitors as in the binding site identification task.

#### 4.3.3 Evaluation metric

This task can be regarded as a binary classification task, whose objective is to classify the match and mismatch patches. Therefore, we follow the settings in Sverrisson *et al.* (2021) to use ROC-AUC as our evaluation metric. Moreover, we also use accuracy, recall, and F1 score as our evaluation metrics.

#### 4.3.4 Experimental results

We present the performance of our method and the competitors in Table 3, where the results of MaSIF are derived from Sverrisson *et al.* (2021). It can be seen that our transformer-based network demonstrates excellent performance in this task, and our self-supervised learning successfully boosts network’s performance in all metrics. Compared to the previous two downstream tasks, pretraining shows marginal improvement on this task because the backbone model already achieves very high performance.

**Table 3.** Performance on protein–protein interaction prediction.

Method	Accuracy↑	Recall↑	F1 score↑	ROC-AUC↑
MaSIF	–	–	–	0.813
dMaSIF	0.795	0.823	0.793	0.862
Ours (from scratch)	0.922	0.990	0.930	0.944
Ours (contrastive)	0.926	**0.994**	0.933	0.945
Ours	**0.927**	**0.994**	**0.934**	**0.948**

We use bold to highlight the optimal value.

### 4.4 Ablation experiment

**Figure 5.**
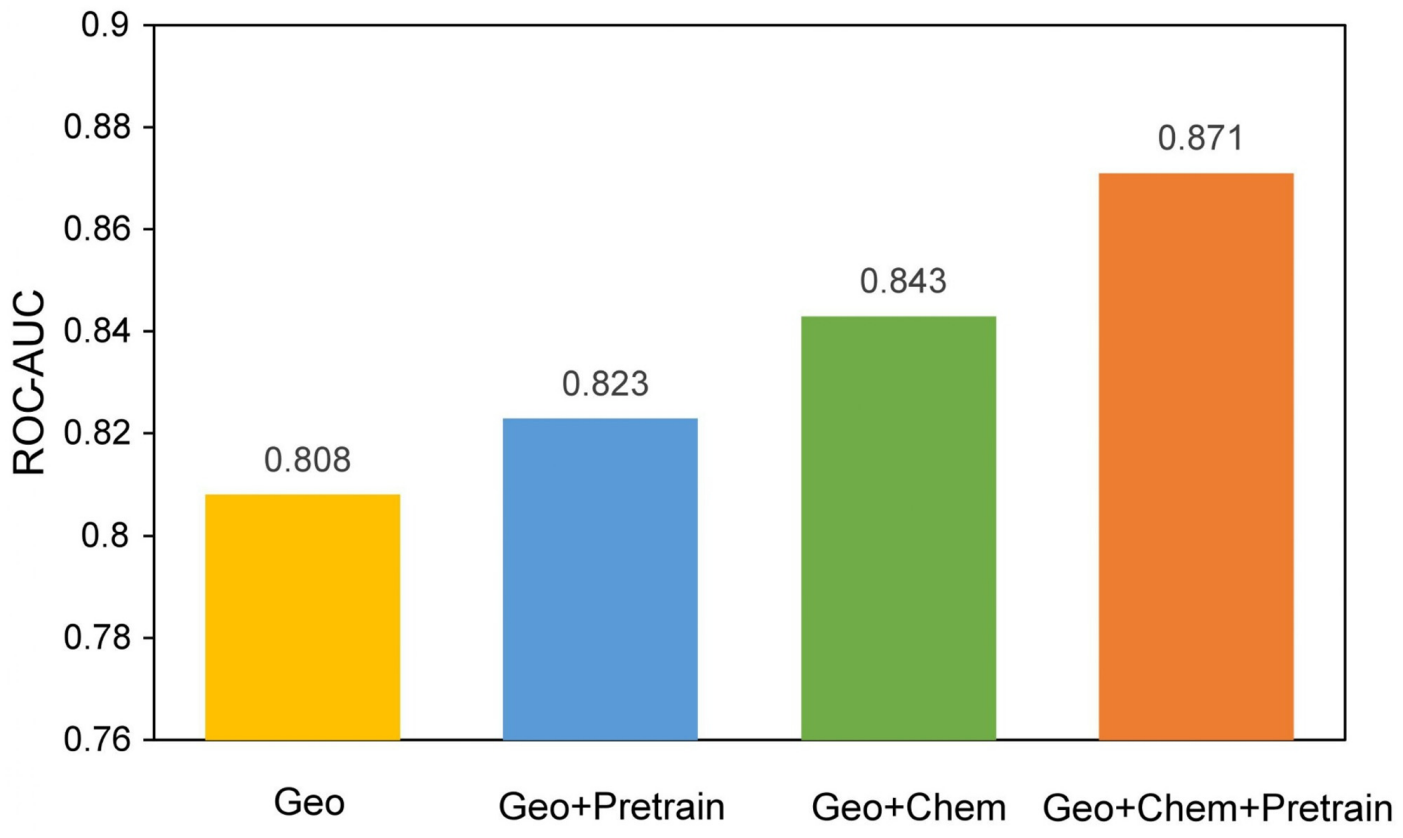
Ablation on chemical features.

We conduct an ablation experiment on the binding site identification task to evaluate importance of chemical features in protein surface representation. The results of this experiment are depicted in Fig. 5. It can be observed that without the chemical features, the ROC-AUC after pretraining significantly decreases (from 0.871 to 0.823). Notably, even without the chemical features, the geometry-based pretraining can still enhance the network’s performance on the downstream task, demonstrating the effectiveness of our self-supervised learning.

### 4.5 Parameter sensitivity analysis

Our self-supervised learning involves the hyper-parameter of mask ratio *m*. To evaluate model’s sensitivity to different mask ratios, we have conducted an experiment to compare the performance of pretraining on the binding site identification task under different mask ratios. As shown in Table 4, our self-supervised learning improves the performance on the downstream task under all mask ratios. This indicates that our self-supervised learning is robust to this parameter. Overall, excessively high or low mask ratios are not conducive for improving the performance on the downstream task.

**Table 4.** Performance on binding site identification task under different mask ratios.

Mask ratio (%)	ROC-AUC ↑
10	0.861
20	0.857
30	0.861
40	0.860
50	0.866
60	**0.871**
70	0.868
80	0.860
90	0.852
From scratch	0.843

We use bold to highlight the optimal value.

### 4.6 Examples of reconstruction

**Figure 6.**
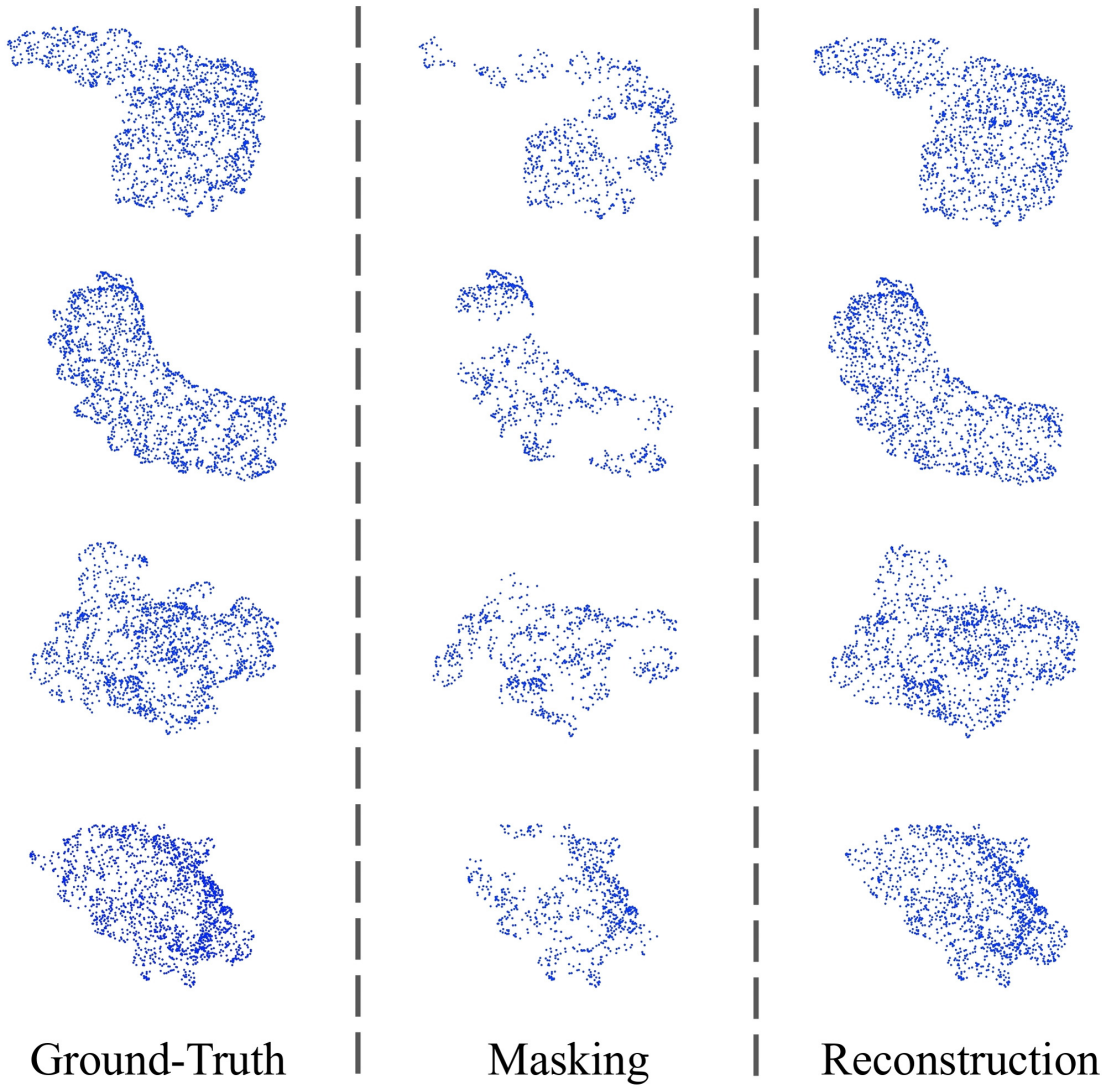
Reconstruction results on pretraining dataset.

We visualize the results of masked reconstruction. As shown in Fig. 6, we visualize several original protein surface point clouds, and the corresponding masked and reconstructed surface point clouds in pretraining. It can be seen that our model can predict the masked parts accurately. We provide more visualizations in Supplementary Material.

### 4.7 Computational efficiency

**Figure 7.**
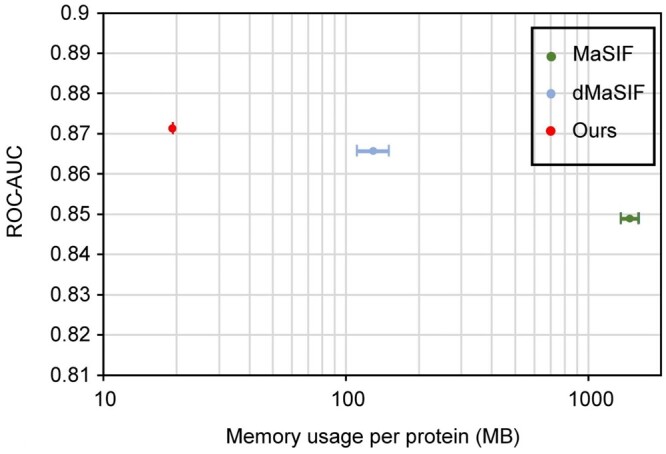
Accuracy (ROC-AUC on binding site identification task) versus memory footprint (MB/protein) of different networks.

Since tasks such as protein design often need implementation on very large datasets, lightweight algorithms can significantly reduce costs. Although previous learning-based methods have shown great advantages in computational cost compared to traditional methods, they still face large computational costs due to the large amount of data. However, our transformer-based network, which applies self-attention at the patch level rather than the point level, has a significant advantage in computational cost over existing surface-based methods. As shown in Fig. 7, our method has a memory cost of about 1/10 of dMaSIF (Sverrisson *et al.* 2021) and 1/100 of MaSIF (Gainza *et al.* 2020), significantly reducing the hardware requirements and saving costs. Moreover, as shown in Table 5, our method also have advantages in inference speed. Both advantageous in memory and speed make our method suitable for practical applications. It is more accessible to researchers and scientists with limited computational resources, enabling them to perform large-scale protein surface analysis tasks more efficiently. This could have significant implications for the field of protein structure prediction and design, as it may allow for more rapid and thorough exploration of protein surfaces, leading to the development of novel protein-based therapeutics, enzymes, and materials.

**Table 5.** Average running time per protein on binding site identification task of different networks.

	MaSIF	dMaSIF	Ours
Time (s/protein)	187.79	0.21	**0.17**

We use bold to highlight the optimal value.

## 5 Conclusion

In this article, we propose a self-supervised learning framework called ProteinMAE to address the issue of data scarcity in protein representation learning. Based on a proxy task of masked reconstruction, we can leverage a large amount of unlabeled data to improve the model’s performance on downstream tasks. To validate its effectiveness, we pretrain a network on the unlabeled PDB dataset and test it on three downstream tasks: binding site identification in protein surface, ligand-binding protein pocket classification, and protein–protein interaction prediction. The results demonstrate that our self-supervised learning not only improves the network’s performance on all downstream tasks but also helps the network achieve competitive performance to state-of-the-art models. Additionally, our ProteinMAE framework is quite efficient, with computational costs far lower than other representation learning methods, making it highly advantageous in practical applications. In the future, we plan to extend this framework to molecular representation learning and explore more downstream tasks.

## Supplementary Material

btad724_Supplementary_Data

## Data Availability

Data and codes in our experiments will be released in https://github.com/phdymz/ProteinMAE.
