# Supplementary material for: ProteinMAE: masked autoencoder for protein surface self-supervised learning
Source: Bioinformatics. 2023 Nov 29;39(12):btad724. doi: 10.1093/bioinformatics/btad724 (PMC10713117; doi:10.1093/bioinformatics/btad724)
Supplement: btad724_Supplementary_Data [file btad724_supplementary_data.pdf]

Supplementary material

1. Data Pre-processing

As depicted in the manuscript, we model the protein surface in terms of point cloud to avoid complex pre-processing. We provide the details of protein surface point cloud construction in this section, which is shown in Figure S1. The proteins with too large size or minority class atoms are filtered, thereby not shown in this figure.

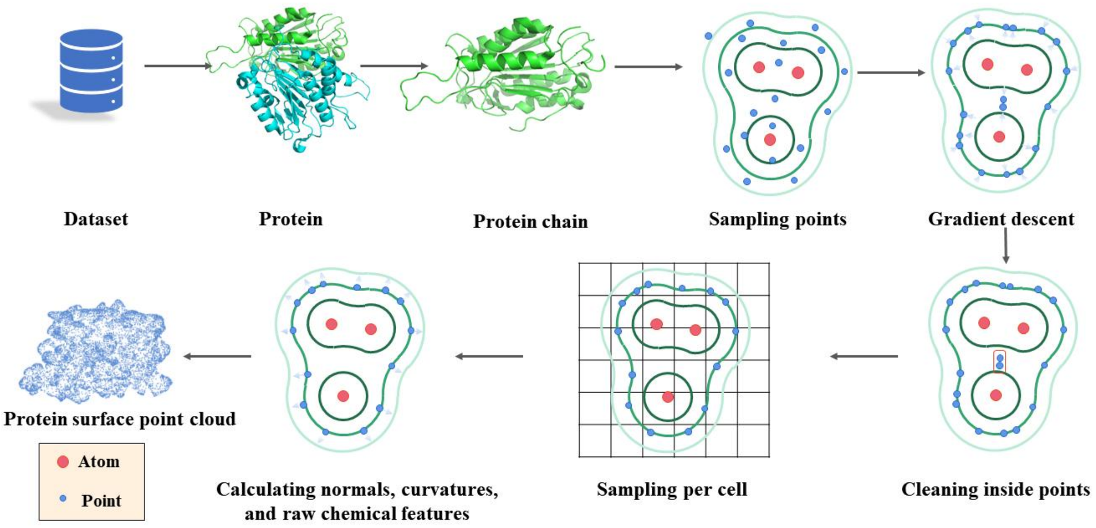

Figure S1. **Pipeline of protein surface point cloud construction.** First, we take protein from the pre-training dataset (downloaded from Protein Data Bank) and decompose it into multiple chains. After that, we utilize the sampling algorithm in dMaSIF to construct the protein surface point cloud for the protein chain. The constructed point cloud not only contains the constructed surface coordinates but also the geometric features (normals and curvatures) and raw chemical features (atom types).

2. Implementation details

In this section, we provide detailed implementations on different downstream tasks.

2.1 Binding site identification in protein surface

Table S1 shows the implementation details on binding site identification task. Figure S2 shows the detailed network architecture for this task.

**Hyper-parameter**	**Settings**
Number of epochs	300
Optimizer	Adam
Learning rate	0.0002
Batch size	32
Number of points of the surface point cloud N	2048
Number of center points g	512
Number of points in each patch k’	32

Table S1. Implementation on binding site identification task.

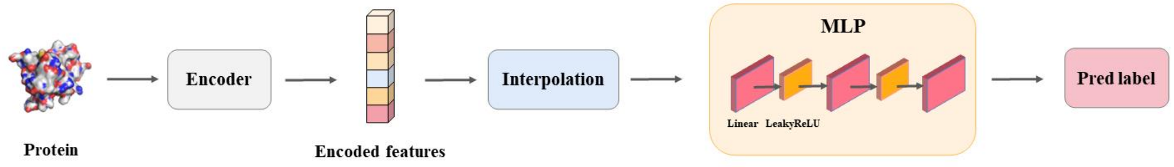

Figure S2. **The network architecture on binding site identification task.** Given an input protein surface point cloud, our encoder will extract patch-level features. Then the extracted features will be interpolated into point-level features. After that, all the point-level features will be fed into an MLP to generate point-level predictions.

2.2 Ligand binding protein pocket classification

Table S2 shows the implementation details on ligand binding protein pocket classification task. Figure S3 shows the detailed network architecture for this task. As shown in Figure S3, the encoder outputs features of CLS token and other tokens. Our head for this task first fuses all the features of all tokens except CLS token and then concatenates the fused feature with CLS token's feature. The concatenated feature is then fed into a MLP to achieve classification.

**Hyper-parameter**	**Settings**
Number of epochs	500
Optimizer	Adam
Learning rate	0.0005
Batch size	32
Number of points of the surface point cloud N	512
Number of center points g	768
Number of points in each patch k'	16

Table S2. Implementation on ligand binding protein pocket classification task.

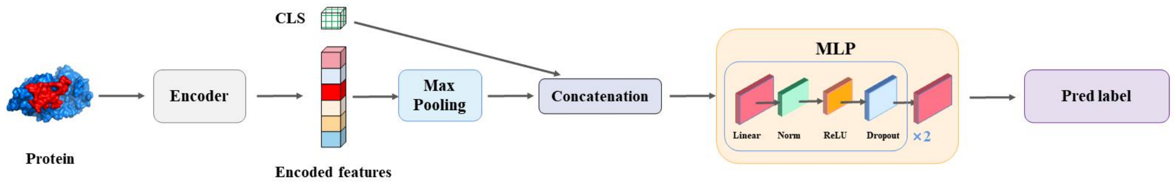

Figure S3. **The network architecture on ligand binding protein pocket classification task.** Given a point cloud of a protein pocket, our encoder will extract features for CLS tokens and other patch tokens. Then the features of patch tokens are aggregated by a max pooling layer. The aggregated features are subsequently concatenated with the feature of CLS token. After that, the concatenated features will be fed into an MLP to generate binding preference to other metabolites.

2.3 Protein-protein interaction prediction

Table S3 shows the implementation details of protein-protein interaction prediction task. Figure S4 shows the detailed network architecture for this task. As depicted in the manuscript, we utilize an identity network as head for this task. Therefore, in this task, we directly utilize the encoded features output by the encoder as the surface descriptors for protein-protein interaction prediction.

**Hyper-parameter**	**Settings**
Number of epochs	500
Optimizer	Adam
Learning rate	0.0005
Batch size	16
Number of points of the surface point cloud N	512
Number of center points g	512
Number of points in each patch k’	16

Table S3. Implementation on protein-protein interaction prediction task.

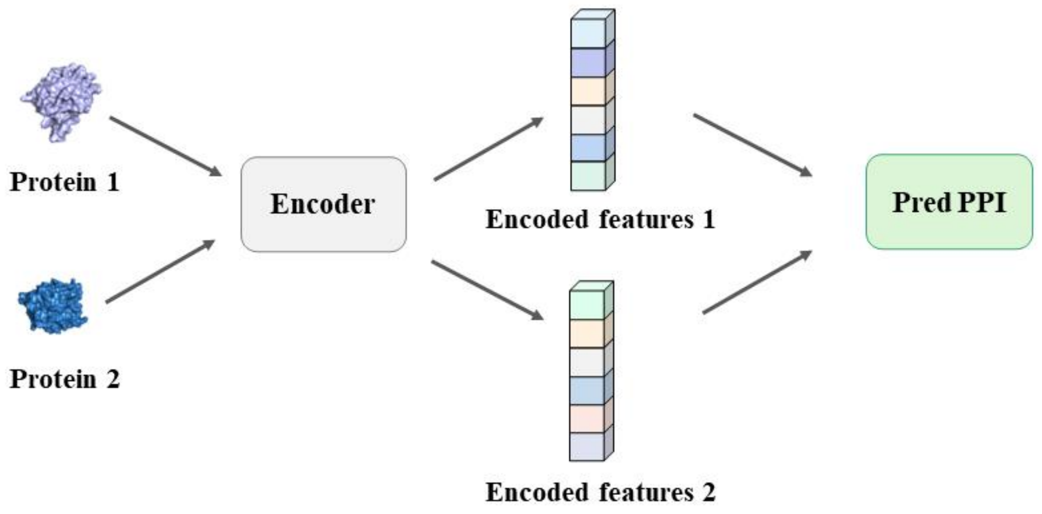

Figure S4. **The network architecture on protein-protein interaction prediction task.** Given two protein surface point clouds, we first utilize encoder to extract patch-level features. Then we predict the probability of their binding based on their feature similarity.

3. Visualization on feature distribution

To further demonstrate the effectiveness of our self-supervised learning qualitatively, we utilize T-SNE [1] to visualize the feature distribution with and without the pre-trained weight initialization. All the features are learned on the ligand binding protein pocket classification task. As shown in Figure S5, with pre-trained weight initialization, the learned features are more separable, which demonstrates the effectiveness of our method intuitively.

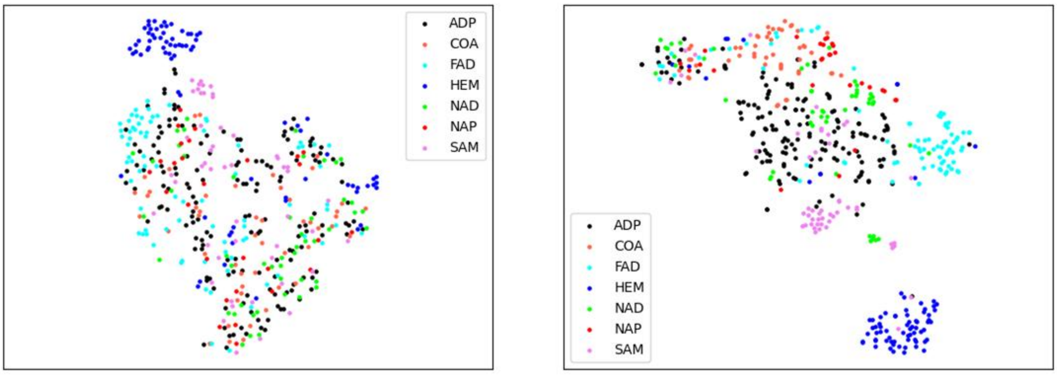

(a) Features trained from scratch

(b) Features fine-tuned

Figure S5. **Visualization on feature distribution.** (a) Features trained from scratch. (b) Features fine-tuned.

4. Dependency on labeled data

As mentioned in the above paragraphs, the acquisition of labeled data such as protein properties is challenging and expensive, because the labels are often obtained through wet experiments. Our self-supervised learning can enhance the performance of downstream tasks using unlabeled data, thus alleviating the dependency on labeled data. To further demonstrate its effectiveness, we conduct an experiment under the different percentages of labeled data. This experiment is conducted on a binding site identification task. The results in Table S4 show that our self-supervised learning can successfully boost the performance on downstream task under different percentages of labeled data and is especially effective when labeled data is extremely scarce.

Percentage of labeled data (%)	From scratch (ROC-AUC↑)	Ours (ROC-AUC↑)
1	0.592	0.713 (+0.121)
2	0.603	0.747 (+0.144)
5	0.717	0.811 (+0.094)
10	0.780	0.829 (+0.049)
20	0.799	0.844 (+0.045)
30	0.816	0.847 (+0.031)
50	0.830	0.863 (+0.033)
100	0.852	0.871 (+0.019)

Table S4. Performance on binding site identification task using different percentage of labeled data.

5. Examples of Reconstruction

We provide more visualization of reconstruction in this section. As shown in Figure S6, the masked proportion can be accurately predicted.

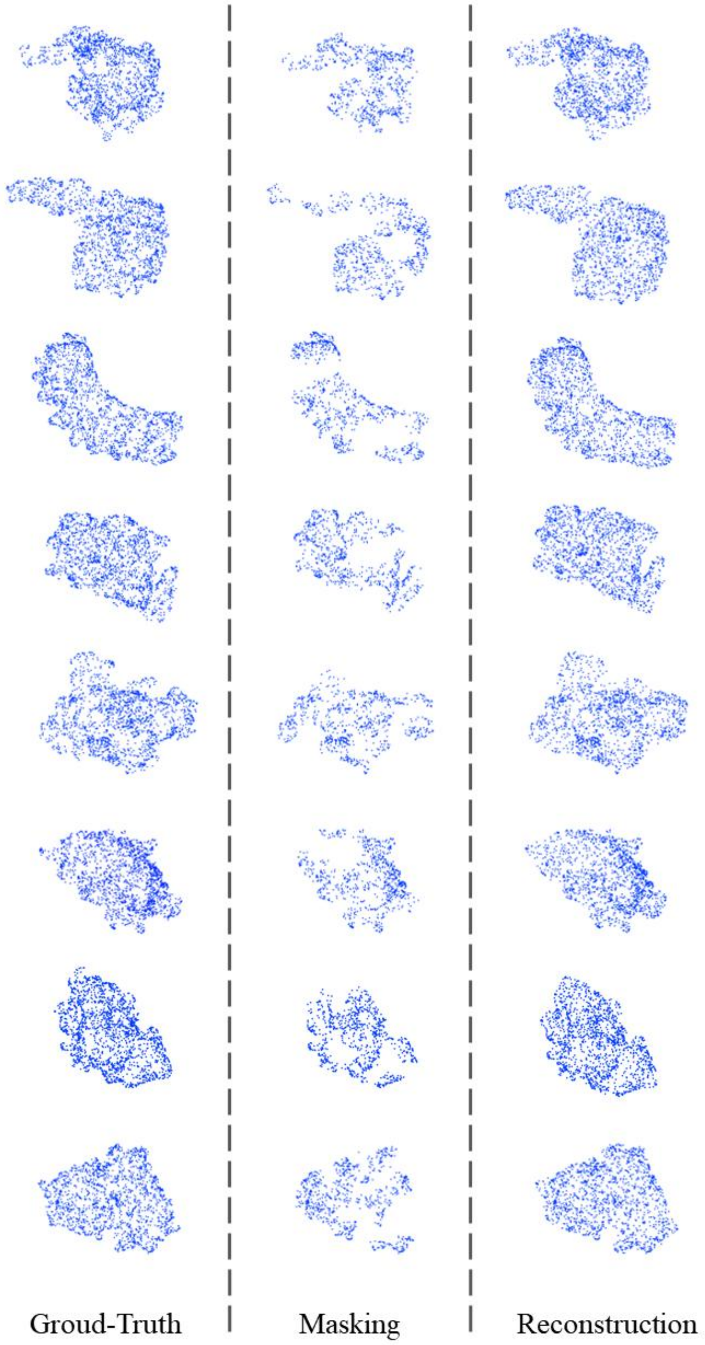

Figure S6. Reconstruction results on pre-training dataset.

Reference:

[1] Van der Maaten L, Hinton G. Visualizing data using t-SNE[J]. Journal of machine learning research, 2008, 9(11).
